# Evaluation of antisense oligonucleotide therapy targeting Hsd17b13 in a fibrosis mice model

**DOI:** 10.1016/j.jlr.2024.100514

**Published:** 2024-02-02

**Authors:** Yanling Ma, Hong Cai, Julia Smith, Ching-Hsuen Chu, Stephen E. Mercer, Stephanie Boehm, Ivar Mcdonald, Bradley Zinker, Dong Cheng

**Affiliations:** Bristol-Myers Squibb Company, Lawrence Township, NJ, USA

**Keywords:** Hsd17b13, antisense oligonucleotide, fibrosis, steatosis, CDAHFD

## Abstract

Human genetic evidence suggests a protective role of loss-of-function variants in 17-beta hydroxysteroid dehydrogenase 13 (HSD17B13) for liver fibrotic diseases. Although there is limited preclinical experimental data on Hsd17b13 antisense oligonucleotide (ASO) or siRNA in a fibrosis model, several ASO and siRNA approaches are being tested clinically as potential therapies for nonalcoholic steatohepatitis (NASH). The aim of this study was to assess the therapeutic potential of Hsd17b13 ASO in a preclinical advanced NASH-like hepatic fibrosis in vivo model. In vitro testing on primary hepatocytes demonstrated that Hsd17b13 ASO exhibited strong efficacy and specificity for knockdown of the Hsd17b13 gene. In choline-deficient, L-amino acid-defined, HFD (CDAHFD)-induced steatotic and fibrotic mice, therapeutic administration of Hsd17b13 ASO resulted in a significant and dose-dependent reduction of hepatic Hsd17b13 gene expression. The CDAHFD group exhibited considerably elevated liver enzyme levels, hepatic steatosis score, hepatic fibrosis, and increased fibrotic and inflammatory gene expression, indicating an advanced NASH-like hepatic fibrosis phenotype. Although Hsd17b13 ASO therapy significantly affected hepatic steatosis, it had no effect on hepatic fibrosis. Our findings demonstrate, for the first time, that Hsd17b13 ASO effectively suppressed Hsd17b13 gene expression both in vitro and in vivo, and had a modulatory effect on hepatic steatosis in mice, but did not affect fibrosis in the CDAHFD mouse model of NASH.

Nonalcoholic steatohepatitis (NASH), a leading cause of liver disease in the general population, is characterized by excessive hepatic fat accumulation with complications of hepatic inflammation, fibrosis, and liver cell damage. Without intervention, NASH could progress into advanced liver diseases, such as cirrhosis and liver cancer, and may even necessitate liver transplantation. Unfortunately, there are currently no Food and Drug Administration-approved drugs for the treatment of NASH; the main disease management strategies rely on lifestyle modifications and the treatment of comorbidities (1).

Accumulating evidence suggests that NASH have a heritable component (2, 3), with genetic variants in several genes, such as *PNPLA3* (4), *TM6SF2* (5), and *MBOAT7* (6), associated with the disease severity. More recently, SNPs within or near the *HSD17B13* gene, which encodes a lipid droplet-associated protein (7, 8, 9) known as hydroxysteroid 17-beta dehydrogenase 13, has also been linked with NASH (10, 11, 12, 13, 14). Specifically, a splice variant (rs72613567) and rs62305723 encoding for a P260S mutation in HSD17B13 are reported to be associated with lower liver enzyme levels and decreased severity of NASH (10). Both splice variants and P260S mutants are enzymatically inactive and/or unstable, leading to the hypothesis that inhibiting HSD17B13 through small-molecule inhibitors or interfering its gene expression through siRNA or antisense oligonucleotide (ASO) might mimic the beneficial effect observed for these variants. In line with this hypothesis, HSD17B13 ASO and siRNA therapy have been developed and are currently undergoing clinical testing in phase I trials for the treatment of NASH.

Despite the enthusiasm for clinical development of HSD17B13-based therapy, preclinical data regarding the pathophysiology of this protein are still limited, and no study has reported the preclinical use of HSD17B13 ASO or siRNA to date. One recent study has evaluated adeno-associated virus (AAV)-mediated Hsd17b13 gene knockdown (KD) using shRNA; however, this approach would not be directly applicable for human use (15). Studies performed in HSD17B13 knockout mice has shown interesting phenotypes that are not entirely consistent with human genetic findings (16). To investigate the therapeutic efficacy of Hsd17b13 ASO on preclinical models, we conducted studies using primary hepatocytes and an in vivo diet-induced NASH-like hepatic fibrosis mouse model. We found that Hsd17b13 ASO robustly inhibited Hsd17b13 gene expression in vitro and in vivo, and therapeutic treatment of Hsd17b13 ASO modulated hepatic steatosis but did not decrease hepatic fibrosis in the rodent NASH model.

## Materials and Methods

### ASOs (A2B5D-038 and A2B5D-039)

Oligonucleotides were synthesized using standard phosphoramidite chemistry on an Äkta Oligopilot 150 oligonucleotide synthesizer. Hsd17b13 ASO had a 5′ to 3′ sequence of **C**G**C**ttttaaggca**C**G**C,** whereas the scramble ASO had a sequence of GG**C**caata**c**gc**c**gT**C**A where capital letters designate locked nucleic acid nucleobases and lowercase letters designate unmodified DNA. Cytosines in bold indicate 5-methylcytosines. The ASOs have full phosphorothioate backbone linkages.

### Animals

Six-week-old C57B6/J male mice were obtained from Jackson Labs (Bar harbor, ME) and were housed two per cage in a temperature- and humidity-controlled environment with free access to food and water. A week later, choline-deficient, l-amino acid-defined, HFD (CDAHFD) (l-amino acid deficient rodent diet with 60 kcal% fat with 0.1% methionine and no added choline, Research Diet, catalog no.: A06071302) was given to 60 mice, whereas 12 mice continue feeding on a control diet (control rodent diet 10 kcal% fat, Research Diet, catalog no.: A13012807) during the full experiment period. Mice on CDAHFD were randomly divided into five groups after the disease induction 4 weeks of diet treatment (*n* = 12) based on body weight and were given saline (vehicle), scramble ASO (50 mpk), or Hsd17b13 ASO at 10, 25, or 50 mpk subcutaneously once a week for 8 consecutive weeks. The mice on control diet were also given saline subcutaneously once a week. Body weight was taken at the start of diet treatment and once a week during drug treatment period. Food intake was measured twice before drug treatment and the last week of drug treatment. All mice were sacrificed 1 week after the last dose, and plasma and tissue sample were collected for further analysis. All experiments were approved by the BMS Animal Care and Use Committee, Lawrenceville, NJ.

### Primary hepatocyte culture and treatment

Mouse primary hepatocytes (Lonza; catalog no.: MBCP01) were thawed and cultured according to the manufacturer’s instruction. Briefly, cells were plated in collagen I-coated 96-well plate (Corning; catalog no.: 356649) at the density of 1.8 × 10^4^ cells/well for 6 h in Lonza maintenance media (MM250-1 and MM250-2). ASOs with concentration ranging from 8.23 nM to 6,000 nM were then added into the wells and incubated with primary hepatocytes for 24–72 h.

### RNA extraction and quantitative PCR

RNA was extracted from mouse liver tissue or primary hepatocytes using MagMAX^TM^-96 Total RNA isolation kit (Life Technologies; catalog no.: AM1830M) and processed on AB Applied Biosystem MagMax express 96 system. Superscript Vilo (Invitrogen; catalog no.: 100011932) was used for complementary DNA synthesis using MJ Research thermal cycler. Real-time quantitative PCR was conducted and analyzed in Bio-Rad CFX 384 Real Time System. Quantitative PCR probes were purchased from Life Technologies with assay IDs listed in Table 1.

**Table 1:** Quantitative PCR probes

Gene name	Quantitative PCR probe assay ID
*Ubc*	Mm02525934_g1
*B2m*	Mm00437762_m1
*Pum1*	Mm01180596_m1
*Rpl13a*	Mm05910660_g1
*Hsd17b13*	Mm01203271_m1
*Cxcl10*	Mm00445235_m1
*Tnfα*	Mm00443258_m1
*Il1b*	Mm00434228_m1
*Il6*	Mm00446190_m1
*Ccl2*	Mm00441244_m1
*Ym1*	Mm00657889_m1
*Gal3*	Mm00802901_m1
*Col1a1*	Mm00801666_g1
*Col1a2*	Mm00483888_m1
*Col3a1*	Mm00802330_m1
*Col4a1*	Mm01210125_m1
*Ctgf*	Mm01192933_g1
*Pai-1(sepine1)*	Mm00435858_m1
*Asma*	Mm007255412_S1
*Mmp7*	Mm00487724_m1
*Mmp12*	Mm00500554_m1
*Tgfb1*	Mm01178820_m1
*Rdh10*	Mm.PT.58.9541215
*Hsd17b11*	Mm.PT.58.42741584
*Dhrs3*	Mm.PT.58.29788869
*Hsd17b6*	Mm.PT.58.43950773

### Plasma marker measurement

Blood samples were collected into EDTA-coated Microvette CB300 tubes, and plasma samples were diluted 2-fold with diH_2_O before measurement. Diluted samples were analyzed using the AU680 Clinical Chemistry Analyzer for alanine transaminase (ALT; Beckman Coulter; catalog no.: OSR6107), aspartate transaminase (AST; Beckman Coulter; catalog no.: OSR6109), alkaline phosphatase (ALP; Beckman Coulter; catalog no.: OSR6004), glucose (Beckman Coulter; catalog no.: OSR), triglyceride (TG; Beckman Coulter; catalog no.: OSR6118), total bile acids (Genway BQ; catalog no.: GWB-BQK086), and NEFA (Wako Diagnostics; catalog no.: NEFA-HR).

### Liver TG measurement

Liver tissue (approximately 100 mg) was homogenized in Dulbecco’s PBS (DPBS) (10 μl/mg tissue) buffer on Precellys Homogenizer. The homogenates were diluted 4-fold in DPBS and analyzed immediately using the AU680 Clinical Chemistry Analyzer for TG (Beckman Coulter; catalog no.: OSR6118).

### Liver histology

The medial and left lateral lobes of the liver were collected, fixed in 10% neutral-buffered formalin, and then embedded in paraffin for histology analysis. Tissue sections were cut onto Superfrost charged slides and stained with H&E for quantification of liver steatosis (steatosis area %) and stained with picrosirius red (PSR) for quantification of liver fibrosis content (area %) using the HALO software (version 2.3; Indica Labs, Inc).

### Liver hydroxyproline measurement

Liver tissue was homogenized in 1 ml DPBS buffer on Precellys Homogenizer and dried in a speed vac for 4 h after adding glacial acetic acid. Samples were hydrolyzed in 1 ml of 6 M HCl solution for 20 h at 110 °C and cooled down to room temperature. Filtered samples were measured for hydroxyproline using Quickzyme Hydroxyproline Kit (Quickzyme; catalog no.: QZBHYPRO5) according to the manufacturer’s instruction.

### Statistical analysis

Nonlinear fit with four parameter variable slope analysis was used for calculation of IC_50_ of Hsd17b13 ASO in primary hepatocytes. One-way AVOVA with Tukey multiple comparisons test was used for difference between treatment groups in vivo.

## Results

### Hsd17b13 ASO effectively inhibited *Hsd17b13* gene expression in primary hepatocytes

**Fig. 1:**
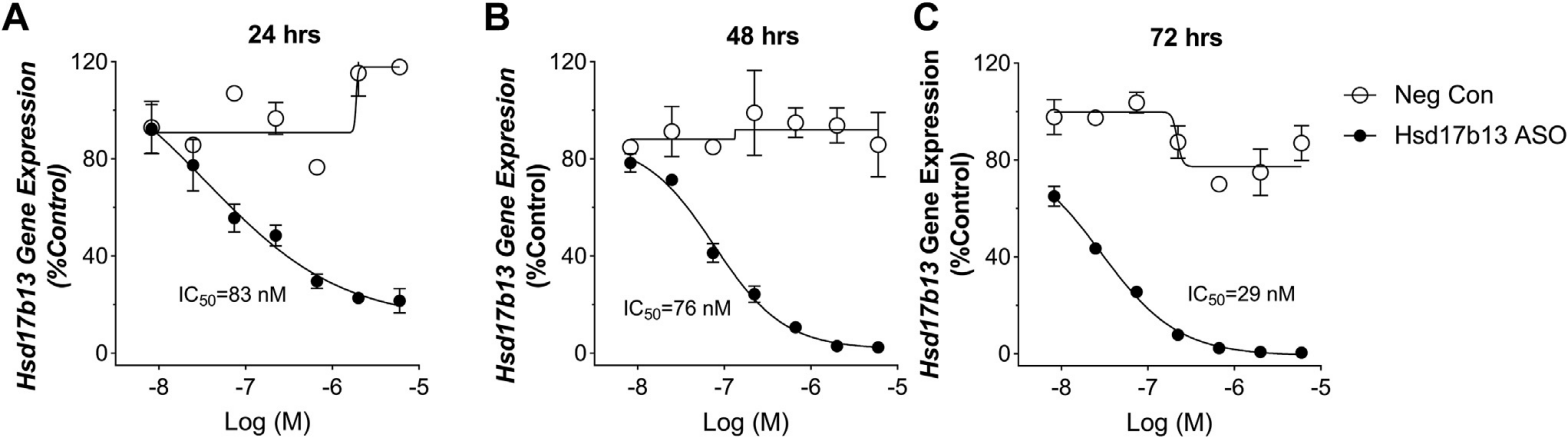
Hsd17b13 ASO effectively inhibited *Hsd17b13* gene expression in primary hepatocytes. Primary mouse hepatocytes were treated with Hsd17b13 ASO (10 nM–6 μM) for (A) 24 h, (B) 48 h, or (C) 72 h. Cellular RNA was extracted and quantified for Hsd17b13 gene expression. Scramble ASO was used as negative control. The IC_50_ of Hsd17b13 ASO was calculated to be 83, 76, and 29 nM at 24, 48, and 72 h, respectively. Data are presented as mean ± SEM.

To determine the KD potency, Hsd17b13 ASO was tested in mouse primary hepatocytes for 24, 48, and 72 h. Negative control scramble ASO had no effect on the gene expression of Hsd17b13, whereas Hsd17b13 ASO effectively eliminated *Hsd17b13* gene expression with an IC_50_ value of 83, 76, and 29 nM, respectively at 24, 48, and 72 h (Fig. 1), suggesting this ASO could efficiently inhibit *Hsd17b13* gene expression in vitro. To further elucidate the specificity of this ASO, gene expression of *Hsd17b11*, *Hsd17b6*, *Rdh10*, and *Dhrs3* was tested in the same experiment setting, and none of these genes were affected by Hsd17b13 ASO.

### Hsd17b13 ASO effectively inhibited *Hsd17b13* gene expression in vivo

**Fig. 2:**
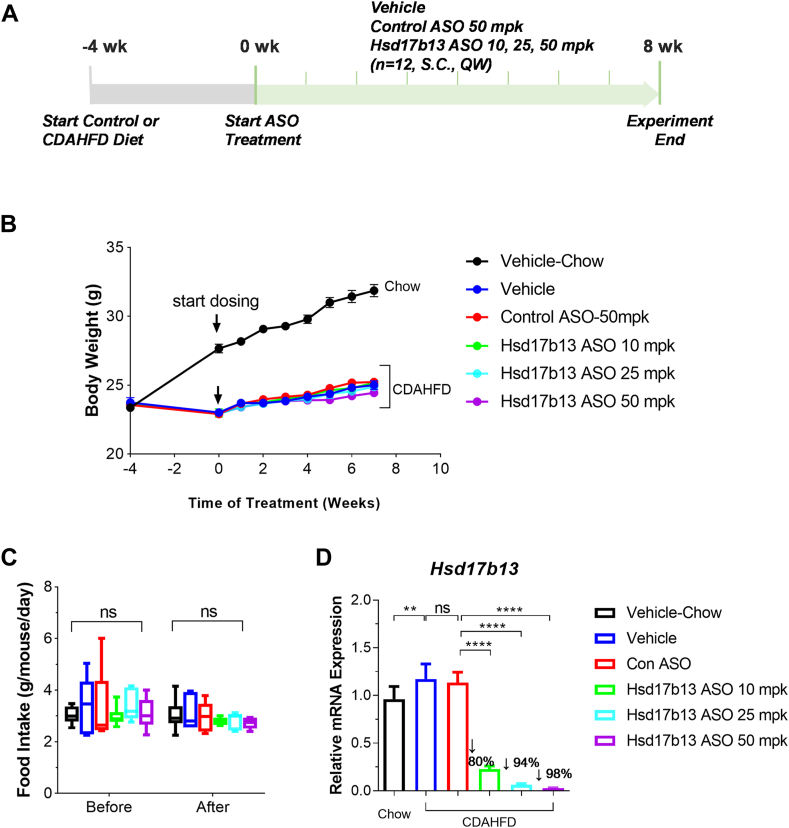
Study design, body weight, food intake, and in vivo KD of *Hsd17b13* by Hsd17b13 ASO. A: Study design. Mice were given control diet or CDAHFD for 4 weeks prior to control or ASO treatment initiation for disease induction. Mice in the control chow diet group were given vehicle; mice in the CDAHFD group were randomly divided into five groups and were given either: vehicle, control ASO at 50 mpk, or Hsd17b13 ASO at 10 mpk, 25 mpk, or 50 mpk, respectively for 8 weeks post disease induction. Vehicle and Hsd17b13 ASOs were administered through subcutaneous injection, once per week. Mice were sacrificed at the end of the experiment and evaluated on NASH markers. B: Body weight was measured once a week during the experiment. C: Food intake prior to respective treatment experiment and at the last week during the experiment. D: Hsd17b13 gene expression of each group at the end of the experiment showing 80%, 94%, and 98% KD of *Hsd17b13* gene by Hsd17b13 ASO at 10, 25, or 50 mpk, respectively. *∗∗P* < 0.01; ∗∗∗∗*P* < 0.0001.

To study the therapeutic potential of Hsd17b13 ASO, we designed an in vivo study on Hsd17b13 ASO using a CDAHFD-induced fibrosis mouse model (Fig. 2A). To mimic human NASH therapy in the clinical setting, the therapeutic treatment, in contrast to preventative treatment, was applied to mice with established fibrosis induced by 4-week CDAHFD treatment. Consistent with previous report (17) mice on CDAHFD maintained their body weight, whereas mice on regular chow diet keep gaining weight steadily during the study (Fig. 2B). There is no significant difference in body weight among groups under CDAHFD diet receiving vehicle, control ASO, or Hsd17b13 ASO at different doses. Food intake was measured before and after ASO treatment, and no difference was found among groups (Fig. 2C). Interestingly, we observed that CDAHFD treatment significantly induced *Hsd17b13* gene expression (Fig. 2D), which is consistent with the clinical observation that HSD17B13 gene expression is elevated in NASH patients compared with healthy control population (10). Control ASO did not change *Hsd17b13* expression as expected. Importantly, as shown in the primary hepatocytes, Hsd17b13 ASO significantly eliminated *Hsd17b13* gene expression by 80%, 94%, and 98%, respectively at 10, 25, and 50 mpk (Fig. 2D) after 8 week treatment.

### Hsd17b13 ASO on plasma markers, liver weight, and liver TG

**Fig. 3:**
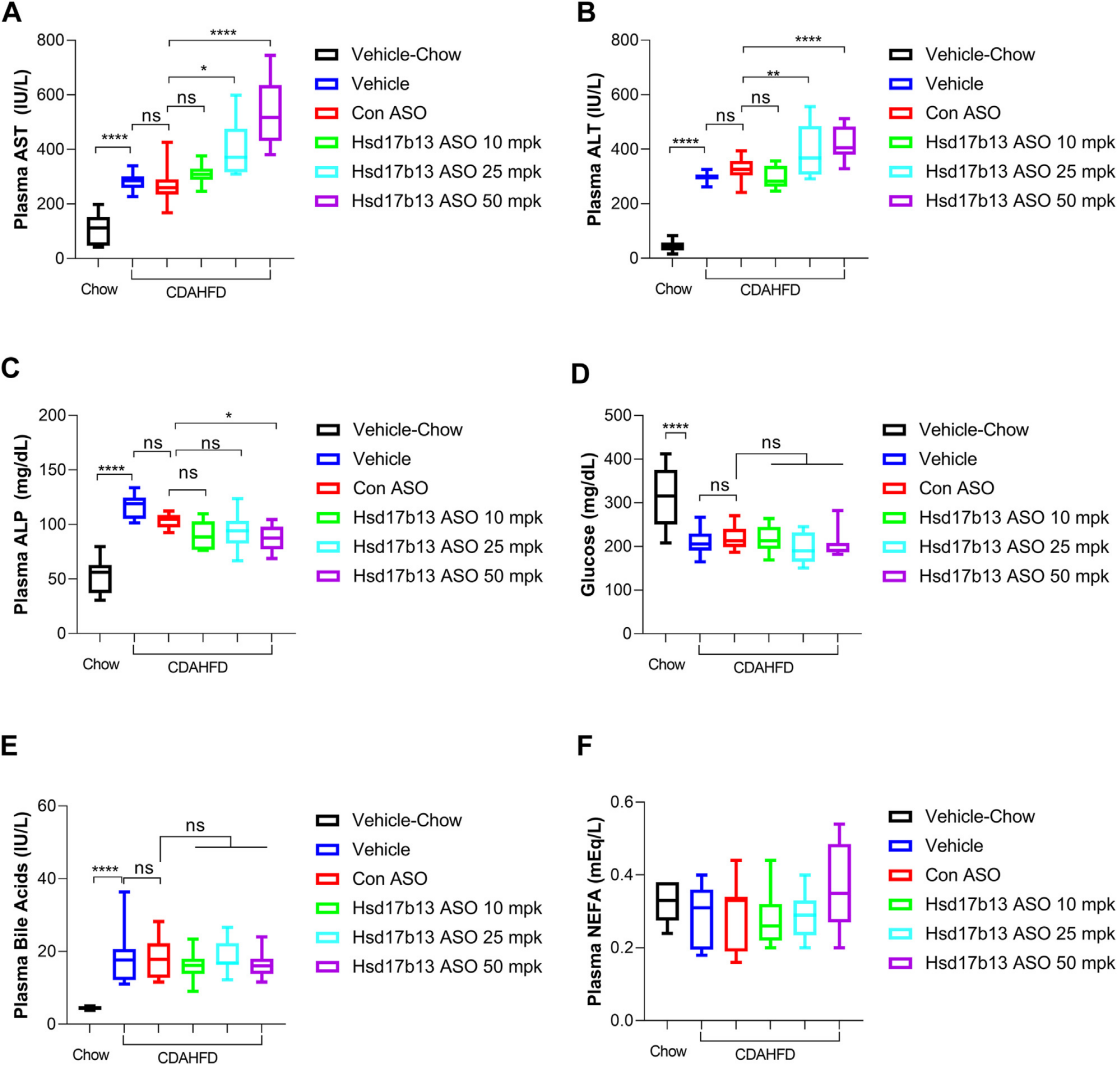
Plasma markers in mice with 8 weeks of vehicle, control ASO, or Hsd17b13 ASO treatment. A: Plasma AST, (B) plasma ALT, (C) plasma ALP, (D) plasma glucose, (E) plasma bile acids, (F) plasma NEFA after 8 weeks of vehicle, control ASO at 50 mpk, or Hsd17b13 ASO at 10 mpk, 25 mpk, or 50 mpk, respectively. ∗*P* < 0.05, ∗∗*P* < 0.01, and ∗∗∗∗*P* < 0.0001.

CDAHFD significantly increased plasma liver enzyme levels (AST, ALT, and ALP, Fig. 3A–C), but decreased blood glucose level (Fig. 3D). As previously described, plasma bile acid level is induced by CDAHFD treatment (18) (Fig. 3E). While Control ASO did not change any of the plasma markers compared with vehicle group, Hsd17b13 ASO at higher doses significantly increased plasma level of AST (Fig. 3A) and ALT (Fig. 3B). The highest dose, 50 mpk group slightly but significantly decreased ALP level (Fig. 3C). No changes were noted in glucose, bile acids, or NEFA by Hsd17b13 ASO treatment compared with control ASO (Fig. 3D–F). A low dose of Hsd17b13 ASO at 10 mpk, however, showed similar plasma markers with control ASO in all respects despite of an 80% KD rate on the Hsd17b13 gene.

**Fig. 4:**
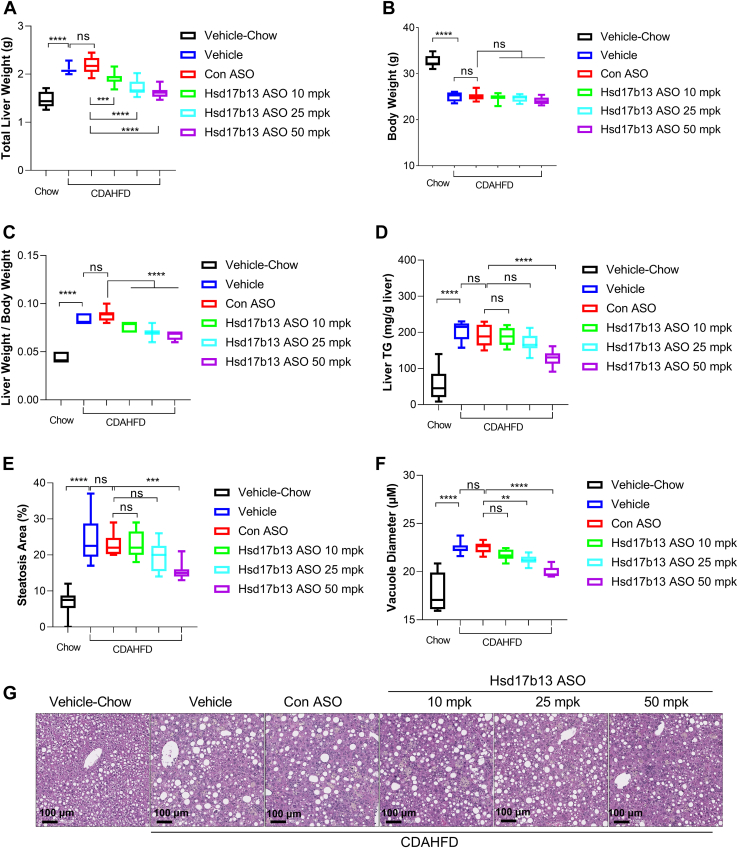
Liver weight, liver histology, and liver TG in mice with 8 weeks of vehicle, control ASO, or Hsd17b13 ASO treatment. A: Mice liver weight after 8 weeks of vehicle, control ASO at 50 mpk, or Hsd17b13 ASO at 10 mpk, 25 mpk, or 50 mpk treatment. B: Mice body weight at the end of the experiment. C: Calculated liver weight to body weight ratio. D: Liver TG content after 8 weeks of vehicle or ASO treatment. E: Steatosis area of mice liver after 8 weeks of vehicle or ASO treatment. F: Lipid vacuole size of mice liver after 8 weeks of vehicle or ASO treatment. G: Representative H&E-stained mice liver tissue after 8 weeks of vehicle or ASO treatment. ∗*P* < 0.05, ∗∗*P* < 0.01, ∗∗∗*P* < 0.001, and ∗∗∗∗*P* < 0.0001.

After 12 weeks of CDAHFD diet, mice showed a significantly increased liver weight (Fig. 4A), decreased body weight (Fig. 4B), and an increased liver weight to body weight ratio (Fig. 4C). The hepatic TG content was significantly elevated in CDAHFD-treated animals compared with the regular chow group (Fig. 4D). Consistently, the mice liver histology and steatosis score evaluation suggested a severe steatosis phenotype under CDAHFD compared with regular chow diet (Fig. 4E–G). Accordingly, the lipid droplet diameter significantly increased in the CDAHFD group as well (Fig. 4F). Control ASO again did not change any of these parameters significantly compared with the vehicle only, whereas Hsd17b13 ASO decreased liver weight, liver weight to body weight ratio, liver TG, liver steatosis, and LD diameter dose-dependently (Fig. 4).

### Therapeutic Hsd17b13 ASO intervention does not affect hepatic fibrosis in mice

**Fig. 5:**
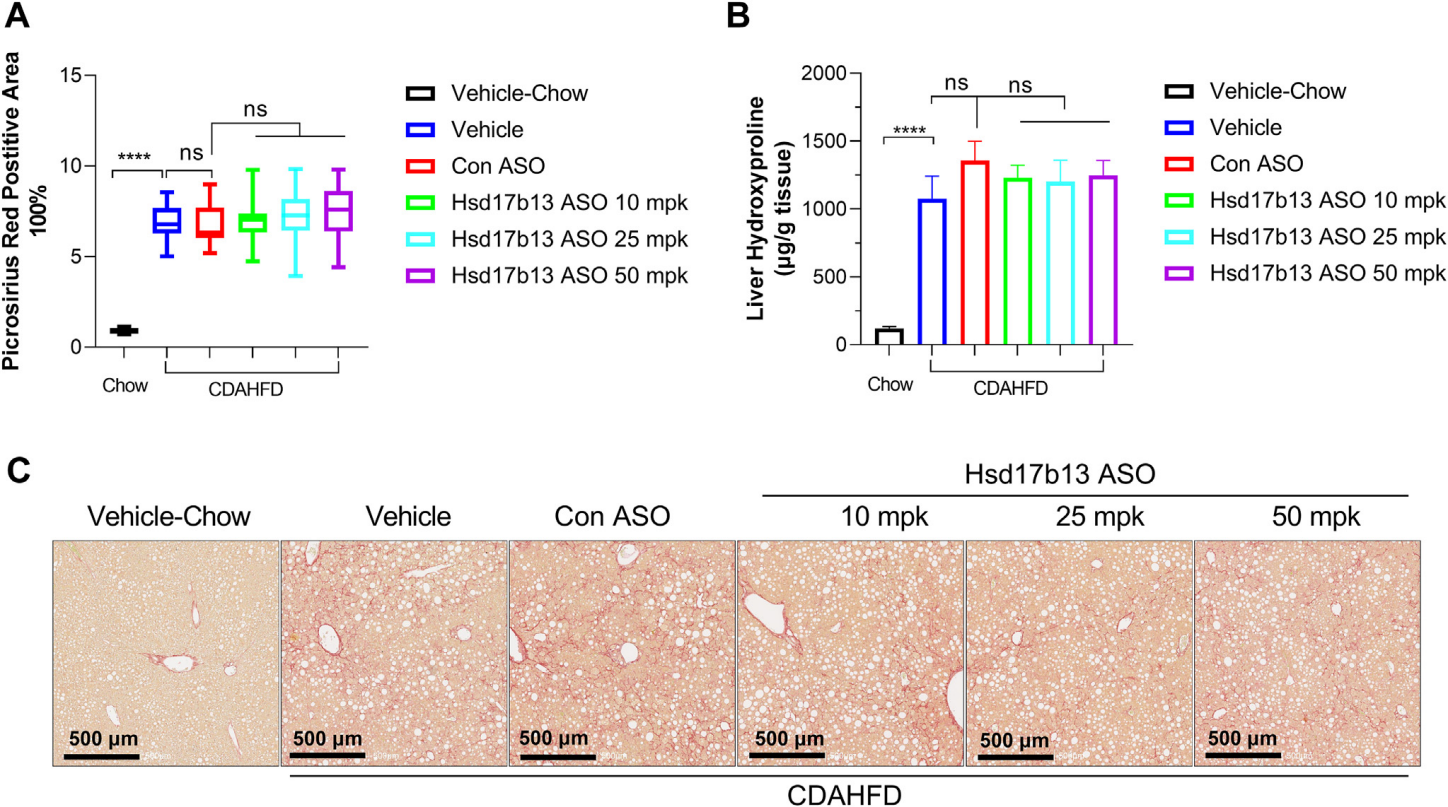
Liver fibrosis evaluation in mice after 8 weeks of vehicle or ASO treatment. A: Quantified PSR-positive staining area. B: Liver hydroxyproline content in mice of each treatment groups (vehicle, control ASO at 50 mpk, or Hsd17b13 ASO at 10 mpk, 25 mpk, or 50 mpk). C: Representative PSR-stained mice liver tissue after 8 weeks of vehicle or ASO treatment. ∗∗∗∗*P* < 0.0001.

**Fig. 6:**
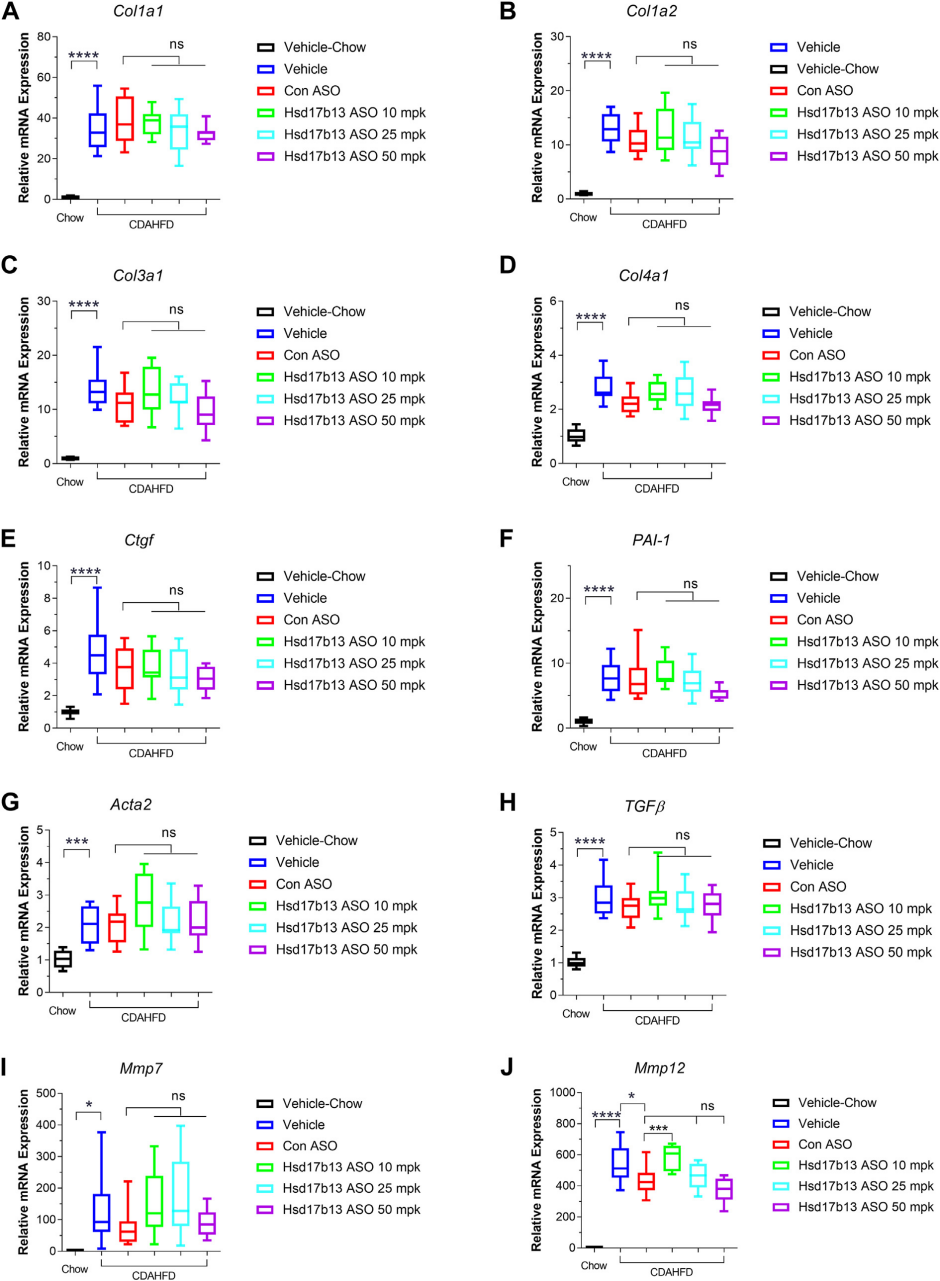
Hepatic fibrotic gene expression in mice with 8 weeks of vehicle or ASO treatment. mRNA was extracted from animals from all groups (vehicle, control ASO at 50 mpk, or Hsd17b13 ASO at 10 mpk, 25 mpk, or 50 mpk), and quantitative PCR was used to determine gene expression of (A) *Col1a1*, (B) *Col1a2*, (C) *Col3a1*, (D) *Col4a1*, (E) *Etgf*, (F) *Pai-1*, (G) *Acta1*, (H) *Tgfb*, (I) Mmp7, and (J) Mmp12. ∗*P* < 0.05, ∗∗*P* < 0.01, and ∗∗∗∗*P* < 0.0001.

**Fig. 7:**
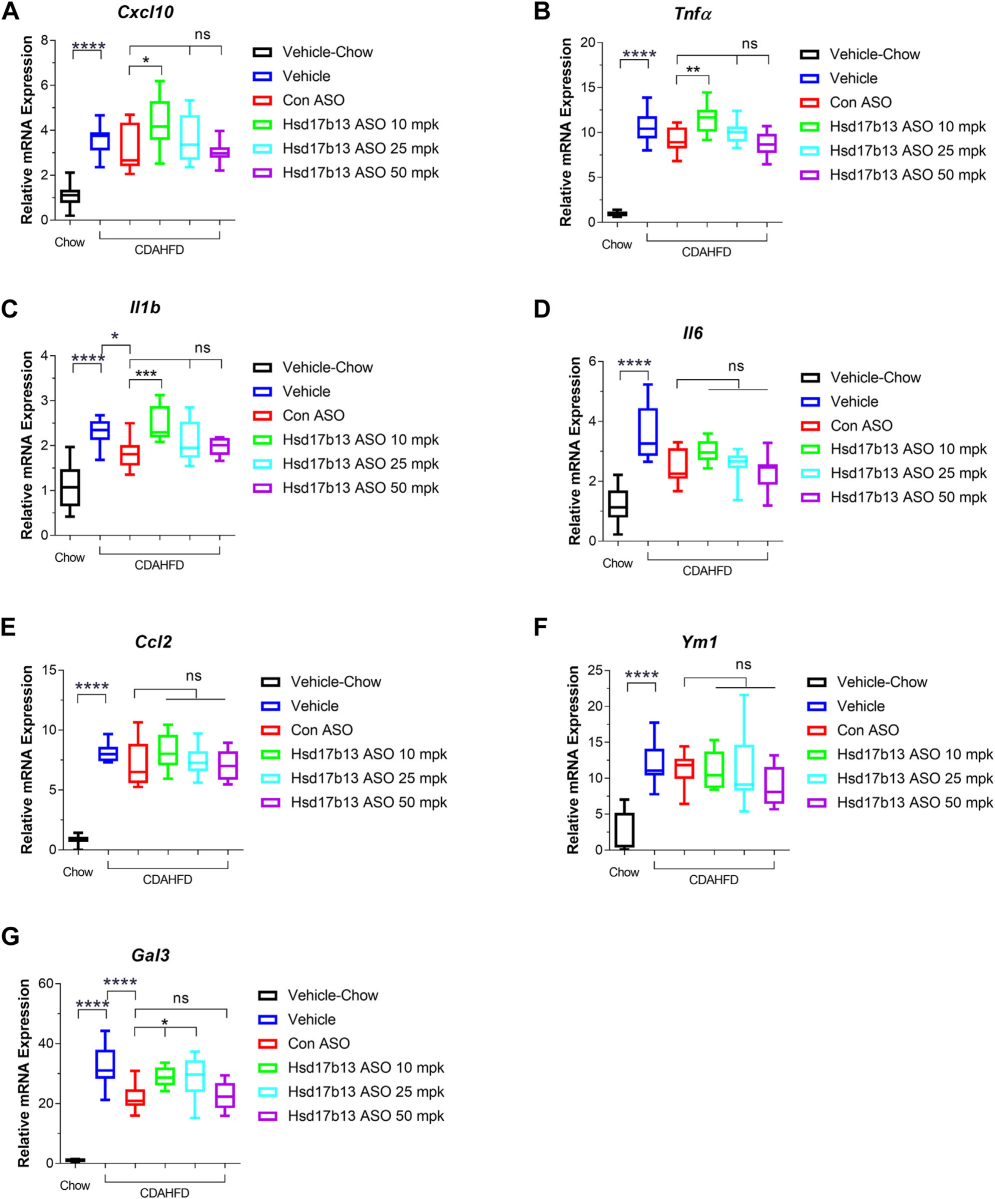
Hepatic inflammatory gene expression in mice with 8 weeks of vehicle or ASO treatment. mRNA was extracted from animals from all groups (vehicle, control ASO at 50 mpk, or Hsd17b13 ASO at 10 mpk, 25 mpk, or 50 mpk), and quantitative PCR was used to determine gene expression of (A) *Cxcl10*, (B) *Tnfα*, (C) *IL1b*, (D) *Il16*, (E) *Ccl2*, (F) *Ym1*, and (G) *Gal3*. ∗*P* < 0.05, ∗∗*P* < 0.01, ∗∗∗*P* < 0.001, and ∗∗∗∗*P* < 0.0001.

We next investigated whether hepatic fibrosis was regulated by Hsd17b13 ASO using PSR staining and gene expression panel analysis. As expected, CDAHFD diet largely induced hepatic fibrosis in mice (Fig. 5). However, therapeutic intervention of Hsd17b13 ASO did not improve hepatic fibrosis. Consistent with this observation, hepatic fibrosis-related genes (*Col1a1*, *Col1a2*, *Col3a1*, *Col4a1*, *Etgf*, *Pai-1*, *Acta1*, *Tgfb*, *Mmp7*, and *Mmp12*) showed no difference between the vehicle and Hsd17b13 ASO-treated groups (Fig. 6). Furthermore, there was no difference in inflammatory-related genes (*Cxcl10*, *Tnfα*, *IL1b*, *Il16*, *Ccl2*, *Ym1*, and *Gal3*) among the vehicle and Hsd17b13 ASO-treated groups (Fig. 7), indicating that therapeutic intervention with Hsd17b13 ASO does not improve liver fibrosis in mice.

## Discussion

Recent human genetic findings that loss-of-function variants in HSD17B13 protect patients from developing severe chronic liver disease have generated enthusiasm of pharmaceutical industry on HSD17B13-based therapy. At least two phase I clinical trials have been conducted or are ongoing to evaluate the safety and tolerability of HSD17B13 ASO or siRNA in humans (19) (https://beta.clinicaltrials.gov/study/NCT05560607; https://beta.clinicaltrials.gov/study/NCT04565717; https://beta.clinicaltrials.gov/study/NCT05519475). However, no experimental data evaluating Hsd17b13 ASO in preclinical fibrosis model have been reported. Data from current study showed that Hsd17b13 ASO effectively and specifically inhibited *Hsd17b13* gene expression in primary hepatocytes, and further in vivo testing confirmed the dose-dependent gene inhibition in fibrotic mice liver. However, therapeutic treatment of Hsd17b13 ASO decreased hepatic steatosis but did not alter the hepatic fibrosis in CDAHFD mice model.

Animal models play a pivotal role in understanding pathophysiology of human diseases and evaluating therapeutic options for human diseases with unmet medical needs (20, 21). However, there are currently no ideal animal models that fully resemble all aspects of NASH characteristics (22). HFD or Western diet-induced obesity models are often used as hepatic steatosis model for NASH, with limited hepatic fibrosis components (16). The CDAHFD model has been well established to represent the fibrosis aspect of human NASH leading to its wide use in academic research and pharmaceutical studies for drug discovery in the NASH field (23). Furthermore, data from previous publications suggest that Hsd17b13 knockout animals showed no protective role in obesogenic diet or alcohol-induced hepatic fibrosis (16). Thus, we decided to use CDAHFD model to better evaluate from hepatic fibrosis perspective. Our data showed that mice developed hepatic steatosis and fibrosis after 12 weeks of CDAHFD, as evidenced by histology, biochemical assays, serum parameters, and hepatic gene expression, suggesting of successful development of NASH-like fibrotic liver diseases in mice. It is important to acknowledge that while the CDAHFD model successfully resembles the hepatic steatosis and fibrosis characteristics of human NASH, like all other diet-induced NASH animal models, it also has its limitations. The CDAHFD mice model does not develop metabolic syndromes that are commonly associated with most human NASH patients. Therefore, this model cannot be used to study the relationship between NASH with metabolic syndromes. In addition, although this model can develop bridging fibrosis and fibrotic nodules if the mice are on the CDAHFD for 26 or more weeks, that was not the case in the current study, as our model was under the CDAHFD for 12 weeks total.

As expected, Hsd17b13 ASO showed robust potency in vitro and in vivo, with KD rate of 80%, 94%, and 98% at 10, 25, and 50 mpk, respectively. Surprisingly, two higher doses showed modulation of liver enzyme levels, suggesting potential safety concerns for use of this ASO in preclinical and clinical settings. It is possible that overinhibiting Hsd17b13 is harmful to the mice liver. We tested specificity of the Hsd17b13 ASO against several Hsd family members (*Hsd17b11*, *Rdh10*, *Dhrs3*, and *Hsd17b6*), and none of them were affected by Hsd17b13 ASO. However, we cannot exclude the possibility that this effect is mediated by off-target effects mediated through other unintended RNA targets that were not included in our study or hybridization-independent toxicity, which requires further investigation as described by Kamola *et al.* (24). Further studies evaluating different Hsd17b13 ASO sequences in a similar experimental setting are also warranted. Besides liver enzymes, all doses of Hsd17b13 ASO had no effect on other serum parameters measured in this study.

It is intriguing that mice treated with Hsd17b13 ASO showed decreased liver weight and hepatic steatosis, consistent with previous reports that Hsd17b13 might regulate hepatic lipid metabolism using genetically modified mouse models. Adam *et al.* (25) reported that Hsd17b13 knockout mice showed increased hepatic steatosis compared with control mice. However, another study demonstrated no overall steatosis but changes in lipid droplet morphology in Hsd17b13 knockout mice under an obesogenic diet (16). Since HSD17B13 has been demonstrated as lipid droplet-associated protein (7, 8), it is plausible that manipulating *Hsd17b13* gene expression by ASO or knockout may play a role in lipid metabolism. Further studies investigating the mechanism between Hsd17b13 and lipid modulation are of great importance to understand the pathophysiology of HSD17B13 and its therapeutic use in humans.

Another interesting finding is that therapeutic treatment with Hsd17b13 ASO did not decrease hepatic fibrosis in CDAHFD mice model at any given dose despite of robust gene inhibition. One might argue that two groups of mice might have been overdosed with Hsd17b13 ASO since they showed increased liver enzyme levels. However, when considering the lowest dose of 10 mpk only, no abnormal liver enzyme level was observed in this group. Nonetheless, there was still no trend of fibrosis improvement measured by PSR staining, hydroxyproline, and fibrotic gene expression, despite of 80% of gene KD, supporting the notion that *Hsd17b13* gene modulation may not affect fibrosis in mice. A previous study discussed the potential interspecies difference between mouse and human HSD17b13 protein, which might explain this unique finding (16). A recent study reported that Hsd17b13 gene silencing by AAV-shRNA induced minor decrease of hepatic fibrosis in mice (15). There are several potential reasons that could explain the differences between our studies. First, Luukkonen *et al.* assessed the preventive effects of Hsd17b13 KD by administering the fibrosis-inducing diet at the same time as AAV injection, whereas we used a more stringent method of inducing disease development in mice for 4 weeks before Hsd17b13 ASO treatment to better mimic the therapeutic process in human NASH. Second, the duration of the treatment may also be accountable for the difference; mice in Luukkonen’s study received AAV-shRNA treatment for 14 weeks, whereas in our study, it was 8 weeks of ASO treatment. Third, although statistically significant, the decrease in fibrosis caused by AAV-shRNA against Hsd17b13 was minor; only a 20% decrease in fibrosis score and ∼10% decrease in liver hydroxyproline was observed in Luukkonen’s study. Notably, we adjusted our unit (to μg/g tissue) to allow for better comparation with Luukkonen’s study data. The Ctrl AAV and Con ASO groups in two studies showed similar level of hepatic hydroxyproline (1,300–1,400 μg/g). In our study, compared with Con ASO, the Hsd17b13 ASO groups showed numerically lower value of hydroxyproline but without statistical significance. Finally, although less likely, we cannot exclude the possibility that the differences caused by diet sources, or the two gene silencing methods of AAV-shRNA and ASO, which warrants further investigations on those interesting findings.

In conclusion, we have tested Hsd7b13 ASO therapy for the first time in a preclinical disease model that parallels that of human NASH and demonstrated that Hsd17b13 intervention disturbed hepatic steatosis but not fibrosis, raising the concern of developing HSD17B13-based therapy for a NASH indication. Further studies should focus on using humanized animal model or alternate ASOs against Hsd17b13 to better shed light on the topic of HSD17B13’s pathophysiological roles in NASH.

## Data availability

All data are contained within the article.

## Conflict of interest

The authors declare that they have no conflicts of interest with the contents of this article.
